# Clinical efficacy and safety of targeted therapy, immunotherapy combined with chemotherapy for treating patients with advanced gastric cancer

**DOI:** 10.12669/pjms.40.9.9049

**Published:** 2024-10

**Authors:** Dongyan Yang, Zhilong Gao, Xuezhu Yang, Liguo Gao

**Affiliations:** 1 Dongyan Yang, Department of Oncology Three Ward, Renmin Hospital, Hubei University of Medicine, Shiyan 442000, China; 2 Zhilong Gao, Department of Gastrointestinal Medicine III, Renmin Hospital, Hubei University of Medicine, Shiyan 442000, China; 3 Xuezhu Yang, Department of Gastroendoscopy, Renmin Hospital, Hubei University of Medicine, Shiyan 442000, China; 4 Liguo Gao, Department of Oncology Three Ward, Renmin Hospital, Hubei University of Medicine, Shiyan 442000, China

**Keywords:** Apatinib, PD-1 inhibitor, Chemotherapy, Advanced gastric cancer

## Abstract

**Objective:**

Exploring the clinical efficacy and safety of targeted therapy, immunotherapy combined with chemotherapy in the treatment of advanced gastric cancer.

**Methods:**

A retrospective analysis was performed on the medical records of 134 patients with advanced gastric cancer who visited Renmin Hospital, Hubei University of Medicine from January 2019 to December 2022. According to therapeutic regimens, enrolled patients were divided into the control group and the study group. Patients in the control group received chemotherapy intervention, while those in the study group were provided with a combined intervention of apatinib, PD-1 inhibitor and chemotherapy. We analyze the tumor control effect and incidence of adverse reactions in two groups of patients.

**Results:**

The disease control rate (DCR) of patients in the study group and the control group was 72.06% and 42.42%, with an overall response rate (ORR) of 8.82% and 4.55%, The differences are statistically significant(P<0.05). By the end of follow-up, the median progression-free survival (mPFS) and the median overall survival (mOS) of the control group patients were 3.0±0.266 and 5.0±0.224 months respectively; while the mPFS and mOS of the study group were 5.0±0.261 and 7.0±0.172 months respectively, the differences are statistically significant (P<0.05). However, there was no significant difference in adverse reactions between the two groups (P>0.05).

**Conclusion:**

The therapeutic regimen of apatinib, a PD-1 inhibitor combined with chemotherapy exhibits relatively high clinical efficacy and safety for the treatment of patients with advanced gastric cancer. It can be considered as an interventional option for this type of patient.

## INTRODUCTION

Gastric cancer is one of the common malignant tumors worldwide. According to epidemiological data in 2020,^1^ its incidence and mortality in China account for approximately 50% of the global incidence and mortality.^2^ Most patients with gastric cancer are already in the advanced stage at the time of treatment, and their prognosis is poor. The 5-year survival rate of stage IV patients is less than 10%. Chemotherapy drugs are still the main treatment for advanced gastric cancer, but the therapeutic effect of traditional chemotherapy is limited. Currently, targeted and immunotherapy have become treatment hotspots. The main measures for the treatment of advanced gastric cancer include chemotherapy, neoadjuvant therapy, radiotherapy and chemoradiotherapy. Significantly, targeted therapy is a targeted drug that specifically acts on specific proteins or pathways of cancer cells. These drugs can inhibit or block key pathways of cancer cell growth and metastasis, thereby inhibiting tumor progression. Immunotherapy is a novel treatment method that has been extensively studied and widely used in various types of tumors.^3^,^4^ The commonly used drugs are clinical immune checkpoint inhibitors, including PD-1/PD-L1 antibodies and CTL-4 antibodies. Related studies have shown that combination therapy is an effective approach for improving the quality of prognosis and prolonging survival in advanced gastric cancer.^5^ However, there is currently limited research on the triple therapy of target immunotherapy combined with chemotherapy for advanced gastric cancer. Therefore, this article explores the clinical efficacy and safety of target immunotherapy combined with chemotherapy in the treatment of advanced gastric cancer, in order to provide reference for the selection of treatment plans for advanced gastric cancer.

## METHODS

A retrospective analysis was performed on the medical records of 134 patients with advanced gastric cancer who visited Renmin Hospital, Hubei University of Medicine from January 2019 to December 2022. According to specific therapeutic regimens, enrolled patients were divided into the control group and the study group. Patients in the control group (N=66) received chemotherapy intervention, while those in the study group (N=68) were provided with a combined intervention of Apatinib, PD-1 inhibitor and chemotherapy. There was no significant difference in baseline characteristics between the two groups of patients (p >0.05), which can meet the needs of subsequent research. The study was approved by the Institutional Ethics Committee of Renmin Hospital, Hubei University of Medicine (No.: syrmyy2023-064; date: October 12, 2023), and written informed consent was obtained from all participants.

### Inclusion criteria:

- Patients with age of ≥18 years old;
- Diagnosed as advanced gastric cancer through imaging and pathological diagnosis;
- With Eastern Cooperative Oncology Group (ECOG) score of 0-2 points;
- With ≥1 measurable lesion(s).

### Exclusion criteria:

- Patients with contraindications to Apatinib, PD-1 inhibitors, or another chemotherapeutics;
- Combined with other malignant tumors;
- Patients with severe organic diseases or congenital diseases (such as valvular heart disease, severe pancreatitis, congenital heart disease, etc.); and incomplete clinical data or lost follow-up.

Patients in the control group received chemotherapy intervention using platinum compounds combined with fluorouracil. The specific regimen included SOX(days 1-14: Gimeracil and Oteracil Potassium Capsules, 40 mg/m^2^, twice a day; and day one: oxaliplatin, 130 mg/m^2^, once per three weeks), XELOX (days 1-14: Capecitabine, 1000 mg/m^2^, twice a day; and day one: oxaliplatin, 130 mg/m^2^, once per three weeks), and FOLFOX (day 1: Calcium folinate, 400 mg/m^2^, fluorouracil, 400 mg/m^2^, and oxaliplatin, 85 mg/m^2^; day two, fluorouracil, 500 mg/m^2^ intravenous infusion, once per two weeks).

Patients in the study group were given targeted therapy, immunotherapy combined with chemotherapy, of which the chemotherapy regimen was the same as that of the control group. The targeted drug was Apatinib (250 mg/500 mg), and the PD-1 inhibitor was Cedilimumab (200 mg, once per three weeks, intravenous infusion for 20-60 min). All patients were followed up via telephone, beginning from the first day of treatment and ending in December 2022.

### Evaluation criterion:

This study recorded and analyzed indicators such as tumor control effect and incidence of adverse reactions in the two groups of patients. The short-term effect was evaluated based on RECIST1.1, it is divided into complete response (CR) (complete disappearance of all lesions, maintenance time ≥4 weeks), partial response (PR) (reduction of lesion length from baseline ≥30%, maintenance time ≥4 weeks), progressive disease (PD) (increase of lesion length from baseline ≥20% or emergence of new lesions), and stable disease (SD) (between PR and PD). Calculate objective response rate (ORR) using CR rate+PR rate, and disease control rate (DCR) using CR rate+PR rate+SD rate, while the long-term effect was assessed using progression- free survival (PFS) and overall survival (OS). Adverse reactions were evaluated according to the Common Terminology Criteria for Adverse Events (CTCAE) 5.0.

### Statistical analysis:

Data analysis of this study employed SPSS26.0. The counting data was represented by frequency, and inter-group comparison was performed using χ^2^ or Fisher’s exact test. Measurement data was represented by the median, and inter-group comparison utilized independent-sample tests. The log-rank test was used for survival comparison. P<0.05 meant that the difference was statistically significant.

## RESULTS

This study included 134 patients with advanced gastric cancer, with 66 cases in the control group and 68 cases in the study group. There was no significant difference in baseline data between the two groups of patients (P>0.05, Table-I). The disease control rate (DCR) in the study group was significantly higher than that in the control group, and PD was significantly lower than that of the latter group (P<0.05). While no significant difference was found in other indicators (P>0.05, Table -II).

**Table-I:** Comparison of baseline data between the two groups of patients with advanced gastric cancer

Baseline data		Control group (N=66)	Study group (N=68)	P
Gender	Male	36 (54.55%)	37 (54.41%)	0.988
	Female	30 (45.45%)	31 (45.59%)	
Age	<65 years old	45 (68.18%)	46 (67.65%)	0.947
	≥65 years old	21 (31.82%)	22 (32.35%)	
Primary tumor site	Diffuse tumor of the whole stomach	11 (16.67%)	8 (11.76%)	0.877
	Cardia and fundus of stomach	17 (25.76%)	19 (27.94%)	
	Gastric body	18 (27.27%)	20 (29.41%)	
	Pylorus and gastric antrum	20 (30.30%)	21 (30.88%)	
ECOG score	0	21 (31.82%)	22 (32.35%)	0.954
	1	32 (48.48%)	34 (50.00%)	
	2	13 (19.70%)	12 (17.65%)	
Lauren’s typing	Intestinal type	19 (28.79%)	18 (26.47%)	0.915
	Mixed type	11 (16.67%)	13 (19.12%)	
	Diffuse type	36 (54.55%)	37 (54.41%)	
BMI	<18.5	21 (31.82%)	23 (33.82%)	0.942
	18.5-23.9	33 (50.00%)	34 (50.00%)	
	≥23.9	12 (18.18%)	11 (16.18%)	
Peritoneal metastasis	With	21 (31.82%)	23 (33.82%)	0.805
	Without	45 (68.18%)	45 (66.18%)	
Ascites	With	19 (28.79%)	20 (29.41%)	0.937
	Without	47 (71.21%)	48 (70.59%)	
Distant metastatic organ	<2	32 (48.48%)	33 (48.53%)	0.996
	≥2	34 (51.52%)	35 (51.47%)	
Chemotherapy regimen	SOX	41 (62.12%)	42 (61.76%)	0.983
	XELOX	16 (24.24%)	16 (23.53%)	
	FOLFOX	9 (13.64%)	10 (14.71%)	

***Note:*** ECOG: Eastern Cooperative Oncology Group; BMI: Body Mass Index.

**Table-II:** Comparison of short-term therapeutic effect between the two groups of patients with advanced gastric cancer.

Effects	Control group (N=66)	Study group (N=68)	X2	P
Complete Response (CR)	0 (0.00%)	0 (0.00%)	-	-
Partial Response (PR)	3 (4.55%)	6 (8.82%)	-	-
Stable Disease (SD)	25 (37.88%)	43 (63.24%)	-	-
Progressive Disease (PD)	38 (57.58%)	19 (27.94%)	-	-
Overall Response Rate (ORR)	3 (4.55%)	6 (8.82%)	0.978	0.323
Disease Control Rate (DCR)	28 (42.42%)	49 (72.06%)	12.033	0.001

By the end of follow-up, the mPFS of the control group patients was 3.0±0.266 months, and the mOS was 5.0±0.224 months; while the mPFS of the study group was 5.0±0.261 months, and the mOS was 7.0±0.172 months. There was significant difference in mPFS and mOS between the two groups of patients (P<0.05, Table-III and Table-IV).

**Table-III:** Mean and median PFS of the two groups of patients.

	Mean^a^				Median			

Groups	Estimate	Standard error	95% confidence interval		Estimate	Standard error	95% confidence interval	
	
			Lower limit	Upper limit			Lower limit	Upper limit
Control study	3.375	0.097	3.185	3.565	3.000	0.266	2.478	3.522
Study group	4.329	0.102	4.129	4.529	5.000	0.261	4.489	5.511
Total	4.047	0.094	3.863	4.231	4.000	0.208	3.593	4.407

**Table-IV:** Mean and median OS of the two groups of patients

	Mean^a^				Median			

Groups	Estimate	Standard error	95% confidence interval		Estimate	Standard error	95% confidence interval	
	
			Lower limit	Upper limit			Lower limit	Upper limit
Control study	5.284	0.101	5.086	5.481	5.000	0.224	4.561	5.439
Study group	6.559	0.088	6.387	6.731	7.000	0.172	6.662	7.338
Total	6.105	0.096	5.916	6.293	6.000	0.195	5.618	6.382

The adverse reactions related to the two groups of patients during treatment were nausea, diarrhea, vomiting, peripheral sensory neuropathy, anemia, fatigue, decreased platelet count, decreased white blood cell count, and decreased neutrophil count. No significant difference was found in adverse reactions between the two groups of patients (P>0.05, Table-V).

**Table-V:** Comparison of adverse reactions between the two groups of patients.

Adverse reactions	Control group (N=66)					Study group (N=68)					P

	Level 1	Level 2	Level 3	Level 4	Level 5	Level 1	Level 2	Level 3	Level 4	Level 5	
Nausea	14	11	0	-	-	11	10	1	-	-	0.543
Diarrhea	7	6	0	0	0	10	8	0	0	0	0.925
Vomiting	7	6	2	0	0	10	5	1	0	0	0.631
Peripheral sensory neuropathy	7	6	2	0	-	9	5	1	0	-	0.714
Anemia	7	5	3	0	0	5	4	1	0	0	0.793
Fatigue	6	6	2	-	-	9	6	0	-	-	0.277
PLT↓	6	5	2	0	-	7	6	1	0	-	0.793
WBC↓	6	5	1	0	-	7	5	1	0	-	0.982
NEUT↓	5	5	2	0	-	6	5	1	0	-	0.809
AST↑	4	5	0	0	-	5	5	1	0	-	0.631
ALT↑	5	4	0	0	-	5	4	1	0	-	0.622

***Note:*** PLT: platelet; WBC: white blood cell; NEUT: Neutrophils; AST: Aspartate transaminase; ALT: Alanine aminotransferase.

## DISCUSSION

This study retrospectively analyzed the combination of apatinib, PD-1 inhibitor, and chemotherapy in the treatment of advanced gastric cancer. The results showed that the DCR of the observation group patients increased to 72.06%, while also prolonging the mFPS and mOS of advanced gastric cancer patients, which is consistent with previous research results. The survival benefits and tumor intervention efficacy of the combination therapy of targeted and immunotherapy are significantly better than other regimens. Our analysis suggests that targeted therapy drugs can quickly control tumor progression, while the tail effect of immunotherapy can bring long-term survival benefits to patients. On the basis of chemotherapy, the combination therapy of targeted and immunotherapy can produce a synergistic effect, thereby improving the survival status of advanced gastric cancer patients.

There are no obvious clinical symptoms in the early stage of gastric cancer.^6^ Most patients, once diagnosed, have progressed to the mid to late stage and cannot receive curative surgical treatment.^7^ There is currently no gold standard for the treatment of advanced gastric cancer, and technologies such as targeted and immunotherapy are the main development directions for the treatment of advanced gastric cancer.^8^,^9^ Multiple studies have confirmed that the application of targeted and immunotherapy regimens can improve the prognosis quality of advanced gastric cancer patients.^10^-^13^ In a single arm prospective phase II trial of advanced gastric cancer, it was shown that the combination treatment of Xindilizumab, Apatinib, and chemotherapy can produce a certain synergistic effect.^14^

During the chemotherapy process for gastric cancer, drug resistance and a series of adverse reactions are prone to occur, leading to a sense of disease uncertainty in patients, which in turn affects their quality of life. This study used a triple regimen of apatinib, PD-1 inhibitor, and chemotherapy to treat advanced gastric cancer. The incidence of adverse reactions during the treatment process was not significantly different from that of a single chemotherapy regimen, indicating its high clinical safety. The reason is that apatinib is a selective targeting tyrosine kinase inhibitor of the novel VEGFR-2 receptor, which has the advantages of high efficiency, low toxicity, good tolerance, and the ability to reverse drug resistance in PTX containing regimens. It has outstanding safety and can significantly improve patient response rates, benefiting the survival of advanced gastric cancer patients.^15^-^18^ Xindilizumab is a recombinant humanized IgG4 anti-PD-1 antibody, and previous studies have shown its clinical safety to be outstanding. Combined chemotherapy can improve the survival of locally advanced gastric cancer patients.^19^,^20^

### Limitations of this study

This study is a retrospective study, with issues such as small sample size and incomplete evaluation. Further prospective research is needed to confirm more convincing results.

## CONCLUSIONS

This study confirms the relatively high clinical efficacy and safety of the therapeutic regimen of Apatinib, PD-1 inhibitor combined with chemotherapy for the treatment of patients with advanced gastric cancer. It can be considered as an interventional option for this type of patients, and further research can be performed for confirmation.

### Authors’ Contributions:

**DY and ZG** carried out the studies data collection, drafted the manuscript, are responsible and accountable for the accuracy or integrity of the work.

**XY** performed the statistical analysis, Review and participated in its design.

**LG** performed the statistical analysis and participated in its design.

All authors have read and approved the final manuscript and are responsible for the accuracy and completeness of the work.
